# Supplementary material for: Reconstructing Prehistoric Viral Genomes from Neanderthal Sequencing Data
Source: Viruses. 2024 May 27;16(6):856. doi: 10.3390/v16060856 (PMC11209150; doi:10.3390/v16060856)
Supplement: Supplementary file 1 [file viruses-16-00856-s001.zip › Supplementary Figure S10.pdf]

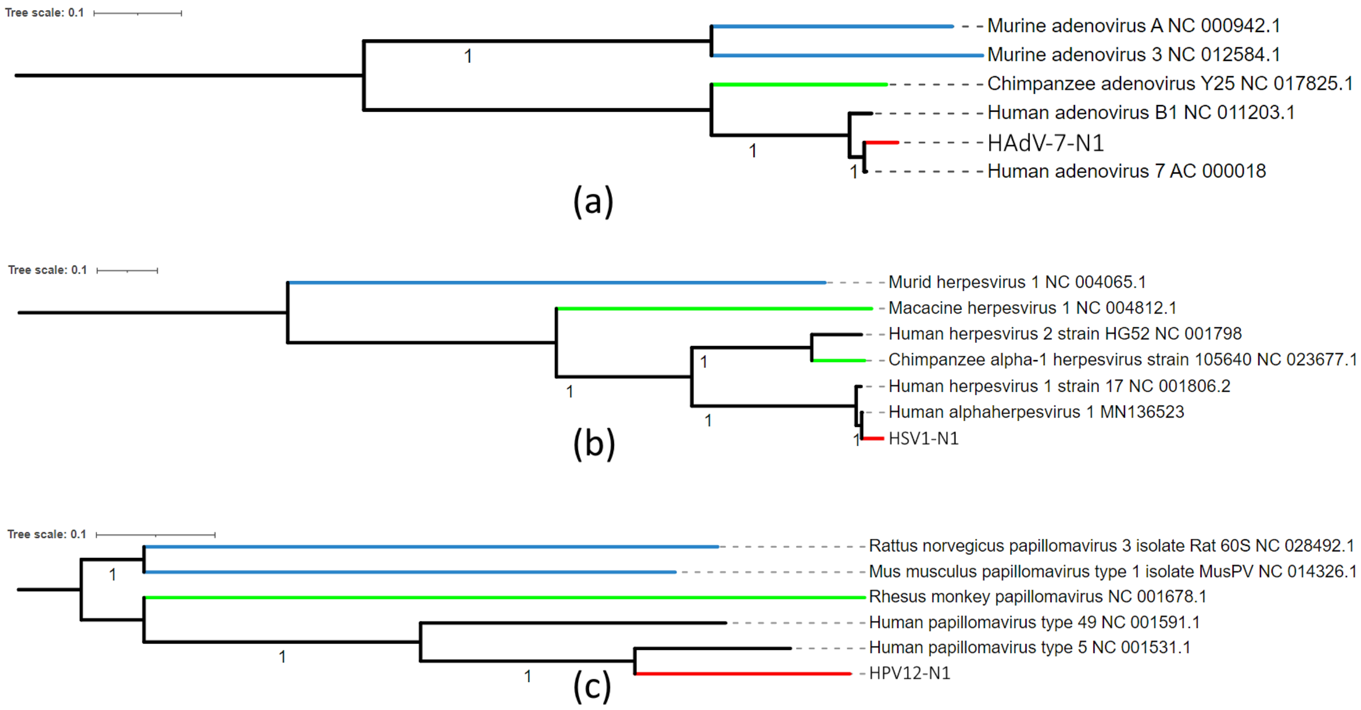

Supplementary Figure S10. Phylogenies of Neanderthal viruses HAdV7-N1 (**a**), HSV1-N1 (**b**) and HPV12-N1 (**c**) compared to NCBI RefSeq primate and murid sequences. Red branches indicate Neanderthal inferred sequences, green branches indicate non-human primate sequences and blue branches indicate murid sequences. Trees were inferred from MAFFT alignments using a Maximum Likelihood model as implemented in FastTree 2.1.11 with GTR model, 4 categories of substitution rates and branch support by Shimodaira-Hasegawa test (Price et al., 2010, *PLoS ONE* 5(3): e9490. https://doi.org/10.1371/journal.pone.0009490). Tree scales (top left of each tree) indicate number of substitutions/sequence position.
