# Supplementary material for: Reconstructing Prehistoric Viral Genomes from Neanderthal Sequencing Data
Source: Viruses. 2024 May 27;16(6):856. doi: 10.3390/v16060856 (PMC11209150; doi:10.3390/v16060856)
Supplement: Supplementary file 1 [file viruses-16-00856-s001.zip › Supplementary Table S1 Adenovirus Modeltest BIC.pdf]

Supplementary Table s1. Parameter estimates by ModelTest-NG and Bayesian Information Criterion for the adenovirus dataset.

BIC	model	K	lnL	score	delta	weight
1	F81+I+G4	5	-55676.6874	113485.8682	0.0000	0.8089
2	HKY+I+G4	6	-55672.9996	113488.9460	3.0778	0.1736
3	TPM2uf+I+G4	7	-55671.2055	113495.8112	9.9430	0.0056
4	TPM3uf+I+G4	7	-55671.2152	113495.8306	9.9624	0.0056
5	TrN+I+G4	7	-55671.3870	113496.1743	10.3061	0.0047
6	TPM1uf+I+G4	7	-55672.8428	113499.0859	13.2177	0.0011
7	TIM2+I+G4	8	-55669.2391	113502.3318	16.4636	0.0002
8	TIM3+I+G4	8	-55669.5951	113503.0439	17.1757	0.0002
9	JC+I+G4	2	-55701.2196	113503.5724	17.7042	0.0001
10	K80+I+G4	3	-55696.8948	113505.3763	19.5081	0.0000

Best model according to BIC

Model:	F81+I+G4
lnL:	-55676.6874
Frequencies:	0.2515 0.2594 0.2542 0.2348
Subst. Rates:	1.0000 1.0000 1.0000 1.0000 1.0000 1.0000
Inv. sites prop:	0.5040
Gamma shape:	0.1924
Score:	113485.8682
Weight:	0.8089

Parameter importances

P.Inv:	-
Gamma:	-
Gamma-Inv:	1.0000
Frequencies:	0.9998

Model averaged estimates

P.Inv:	-
Alpha:	-
Alpha-P.Inv:	0.2345
P.Inv-Alpha:	0.5312
Frequencies:	0.2515 0.2594 0.2542 0.2348
