# Supplementary material for: Reconstructing Prehistoric Viral Genomes from Neanderthal Sequencing Data
Source: Viruses. 2024 May 27;16(6):856. doi: 10.3390/v16060856 (PMC11209150; doi:10.3390/v16060856)
Supplement: Supplementary file 1 [file viruses-16-00856-s001.zip › Supplementary Table S10.pdf]

Supplementary Table 10. Similarity matrix of human, primate, and murid adenovirus NCBI RefSeq sequences.

Adenovirus sequences	Human adenovirus 7 AC_000018	HAdV-7-N1 consensus	Human adenovirus B1 NC_011203.1	Chimpanzee adenovirus Y25 NC_017825.1	Murine adenovirus 3 NC_012584.1	Murine adenovirus A NC_000942.1
Human adenovirus 7 AC_000018	100.0	94.7	95.3	71.3	40.4	41.3
HAdV-7-N1 Consensus	94.7	100.0	92.0	69.5	40.0	40.8
Human adenovirus B1 NC_011203.1	95.3	92.0	100.0	71.6	40.4	41.4
Chimpanzee adenovirus Y25 NC_017825.1	71.3	69.5	71.6	100.0	39.0	40.0
Murine adenovirus 3 NC_012584.1	40.4	40.0	40.4	39.0	100.0	64.4
Murine adenovirus A NC_000942.1	41.3	40.8	41.4	40.0	64.4	100.0
