# Supplementary material for: Reconstructing Prehistoric Viral Genomes from Neanderthal Sequencing Data
Source: Viruses. 2024 May 27;16(6):856. doi: 10.3390/v16060856 (PMC11209150; doi:10.3390/v16060856)
Supplement: Supplementary file 1 [file viruses-16-00856-s001.zip › Supplementary Table S11.pdf]

Supplementary Table 11. Identity (%) matrix of human, primate, and murid herpesvirus NCBI RefSeq sequences.

Herpesvirus sequences	HSV1-N1 consensus	Human alphaherpesvirus 1 MN136523	Human herpesvirus 1 strain 17 NC_001806.2	Chimpanzee alpha-1 herpesvirus strain 105640 NC_023677.1	Human herpesvirus 2 strain HG52 NC_001798	Macacine herpesvirus 1 NC_004812.1	Murid herpesvirus 1 NC_004065.1
HSV1-N1 consensus	100.0	95.7	94.5	70.5	70.1	56.6	28.6
Human alphaherpesvirus 1 MN136523	95.7	100.0	97.7	72.1	71.8	57.9	29.0
Human herpesvirus 1 strain 17 NC_001806.2	94.5	97.7	100.0	72.3	72.1	57.8	29.0
Chimpanzee alpha-1 herpesvirus strain 105640 NC_023677.1	70.5	72.1	72.3	100.0	88.4	58.2	29.2
Human herpesvirus 2 strain HG52 NC_001798	70.1	71.8	72.1	88.4	100.0	59.0	29.5
Macacine herpesvirus 1 NC_004812.1	56.6	57.9	57.8	58.2	59.0	100.0	29.6
Murid herpesvirus 1 NC_004065.1	28.6	29.0	29.0	29.2	29.5	29.6	100.0
