# Supplementary material for: Reconstructing Prehistoric Viral Genomes from Neanderthal Sequencing Data
Source: Viruses. 2024 May 27;16(6):856. doi: 10.3390/v16060856 (PMC11209150; doi:10.3390/v16060856)
Supplement: Supplementary file 1 [file viruses-16-00856-s001.zip › Supplementary Table S12.pdf]

Supplementary Table 12. Identity (%) matrix of human, primate, and murid papillomavirus NCBI RefSeq sequences.

Papillomavirus Sequences	HPV12-N1 consensus	Human papillomavirus type 5 NC_001531.1	Human papillomavirus type 49 NC_001591.1	*Mus musculus* papillomavirus type 1 isolate MusPV NC_014326.1	*Rattus norvegicus* papillomavirus 3 isolate Rat_60S NC_028492.1	*Rhesus* monkey papillomavirus NC_001678.1
HPV12-N1 consensus	100.0	74.0	59.3	41.9	42.9	39.7
Human papillomavirus type 5 NC_001531.1	74.0	100.0	61.1	42.4	44.0	40.1
Human papillomavirus type 49 NC_001591.1	59.3	61.1	100.0	43.2	44.5	41.1
Mus musculus papillomavirus type 1 isolate MusPV NC_014326.1	41.9	42.4	43.2	100.0	43.7	39.4
Rattus norvegicus papillomavirus 3 isolate Rat_60S NC_028492.1	42.9	44.0	44.5	43.7	100.0	39.7
Rhesus monkey papillomavirus NC_001678.1	39.7	40.1	41.1	39.4	39.7	100.0
