# Supplementary material for: Reconstructing Prehistoric Viral Genomes from Neanderthal Sequencing Data
Source: Viruses. 2024 May 27;16(6):856. doi: 10.3390/v16060856 (PMC11209150; doi:10.3390/v16060856)
Supplement: Supplementary file 1 [file viruses-16-00856-s001.zip › Supplementary Table S2 Herpesvirus Modeltest BIC.pdf]

Supplementary Table S2. Parameter estimates by ModelTest-NG and Bayesian Information Criterion for the herpesvirus dataset.

BIC	model	K	lnL	score	delta	weight
1	GTR+I+G4	10	-370860.6877	744165.4747	0.0000	0.9905
2	TVM+I+G4	9	-370871.2967	744174.7703	9.2956	0.0095
3	TIM1+I+G4	8	-370961.0703	744342.3950	176.9202	0.0000
4	TPM1uf+I+G4	7	-370970.8451	744350.0223	184.5475	0.0000
5	TIM3+I+G4	8	-371646.0510	745712.3565	1546.8817	0.0000
6	TPM3uf+I+G4	7	-371659.9747	745728.2815	1562.8067	0.0000
7	TIM2+I+G4	8	-371764.7860	745949.8265	1784.3518	0.0000
8	TPM2uf+I+G4	7	-371772.1024	745952.5367	1787.0620	0.0000
9	TrN+I+G4	7	-371985.5530	746379.4379	2213.9632	0.0000
10	HKY+I+G4	6	-371994.2478	746384.9051	2219.4304	0.0000

Best model according to BIC

Model:	GTR+I+G4
lnL:	-370860.6877
Frequencies:	0.1599 0.3371 0.3438 0.1592
Subst. Rates:	0.9903 1.8152 0.6142 0.3307 2.0155 1.0000
Inv. sites prop:	0.8408
Gamma shape:	0.2714
Score:	744165.4747
Weight:	0.9905

Parameter importances

P.Inv:	-
Gamma:	-
Gamma-Inv:	1.0000
Frequencies:	1.0000

Model averaged estimates

P.Inv:	-
Alpha:	-
Alpha-P.Inv:	0.2714
P.Inv-Alpha:	0.8408
Frequencies:	0.1599 0.3371 0.3438 0.1592
