# Supplementary material for: Reconstructing Prehistoric Viral Genomes from Neanderthal Sequencing Data
Source: Viruses. 2024 May 27;16(6):856. doi: 10.3390/v16060856 (PMC11209150; doi:10.3390/v16060856)
Supplement: Supplementary file 1 [file viruses-16-00856-s001.zip › Supplementary Table S3 Papillomavirus Modeltest BIC.pdf]

Supplementary Table S3. Parameter estimates by ModelTest-NG and Bayesian Information Criterion for the papillomavirus dataset.

BIC	model	K	lnL	score	delta	weight
1	GTR+I+G4	10	-58362.0041	118089.9482	0.0000	1.0000
2	TIM2+I+G4	8	-58383.9226	118115.9297	25.9815	0.0000
3	TVM+I+G4	9	-58379.6185	118116.2493	26.3011	0.0000
4	TPM2uf+I+G4	7	-58397.0877	118133.3322	43.3840	0.0000
5	TIM3+I+G4	8	-58393.3002	118134.6849	44.7367	0.0000
6	TPM3uf+I+G4	7	-58406.7987	118152.7542	62.8060	0.0000
7	TrN+I+G4	7	-58410.1869	118159.5307	69.5825	0.0000
8	TIM1+I+G4	8	-58406.0633	118160.2111	70.2629	0.0000
9	HKY+I+G4	6	-58419.6900	118169.6090	79.6609	0.0000
10	TPM1uf+I+G4	7	-58415.6606	118170.4781	80.5299	0.0000

Best model according to BIC

Model:	GTR+I+G4
lnL:	-58362.0041
Frequencies:	0.3216 0.1878 0.2134 0.2772
Subst. Rates:	1.9422 3.8666 1.6240 1.6263 5.2034 1.0000
Inv. sites prop:	0.3572
Gamma shape:	1.0434
Score:	118089.9482
Weight:	1.0000

Parameter importances

P.Inv:	-
Gamma:	-
Gamma-Inv:	1.0000
Frequencies:	1.0000

Model averaged estimates

P.Inv:	-
Alpha:	-
Alpha-P.Inv:	1.0434
P.Inv-Alpha:	0.3572
Frequencies:	0.3216 0.1878 0.2134 0.2772
