# Supplementary figures and images for: Reconstructing Prehistoric Viral Genomes from Neanderthal Sequencing Data
Source: Viruses. 2024 May 27;16(6):856. doi: 10.3390/v16060856 (PMC11209150; doi:10.3390/v16060856)

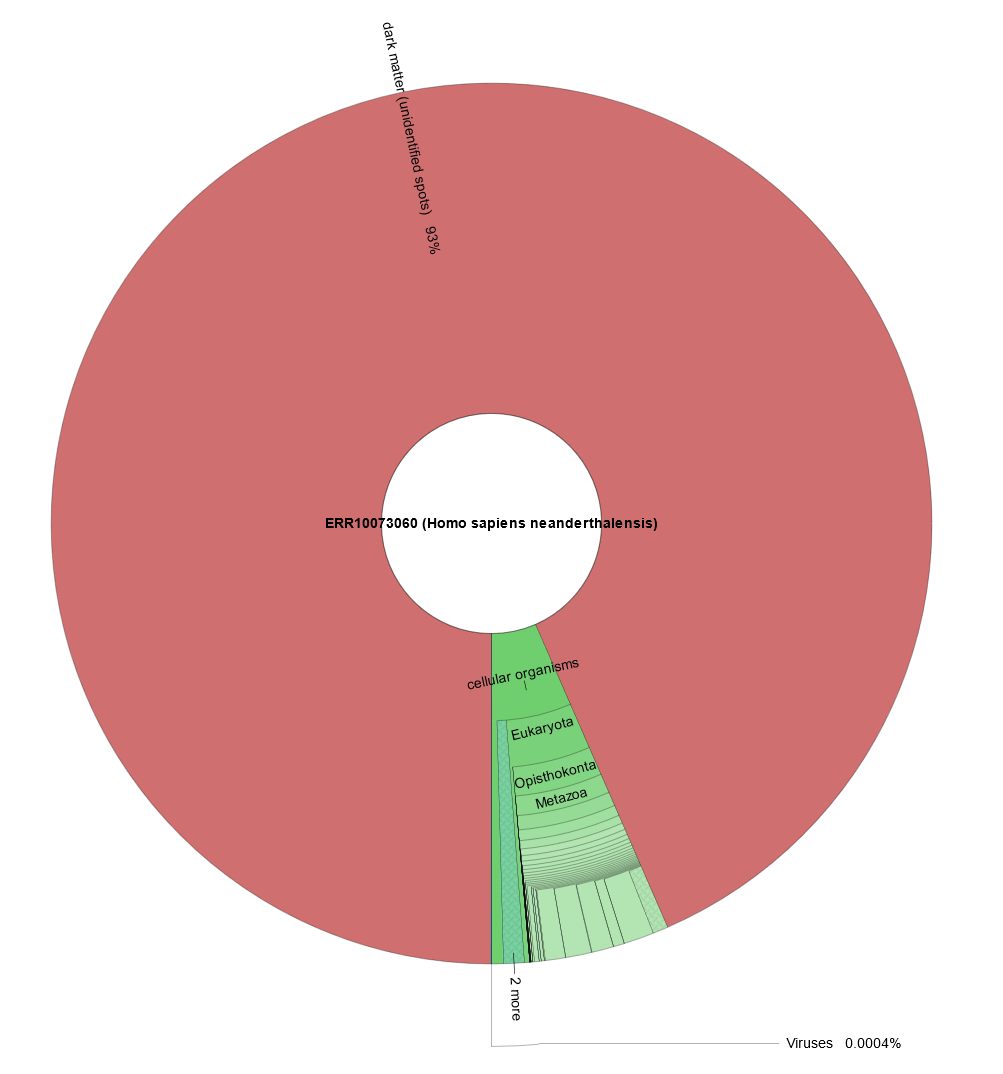

Supplement: Supplementary file 1 [file viruses-16-00856-s001.zip › Supplementary Data S17 - Krona view of ERR10073060 Run.png]

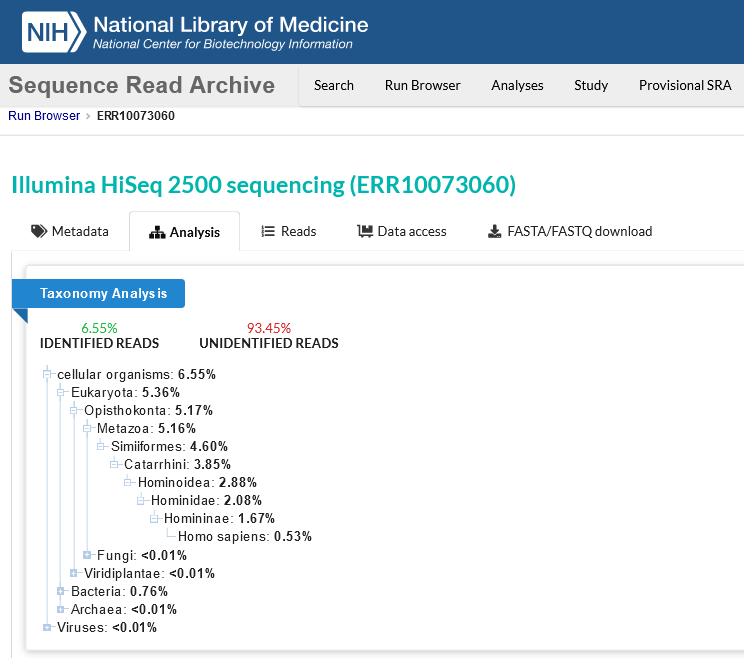

Supplement: Supplementary file 1 [file viruses-16-00856-s001.zip › Supplementary Data S18 - ERR10073060 Run Browser SRA Archive NCBI.png]

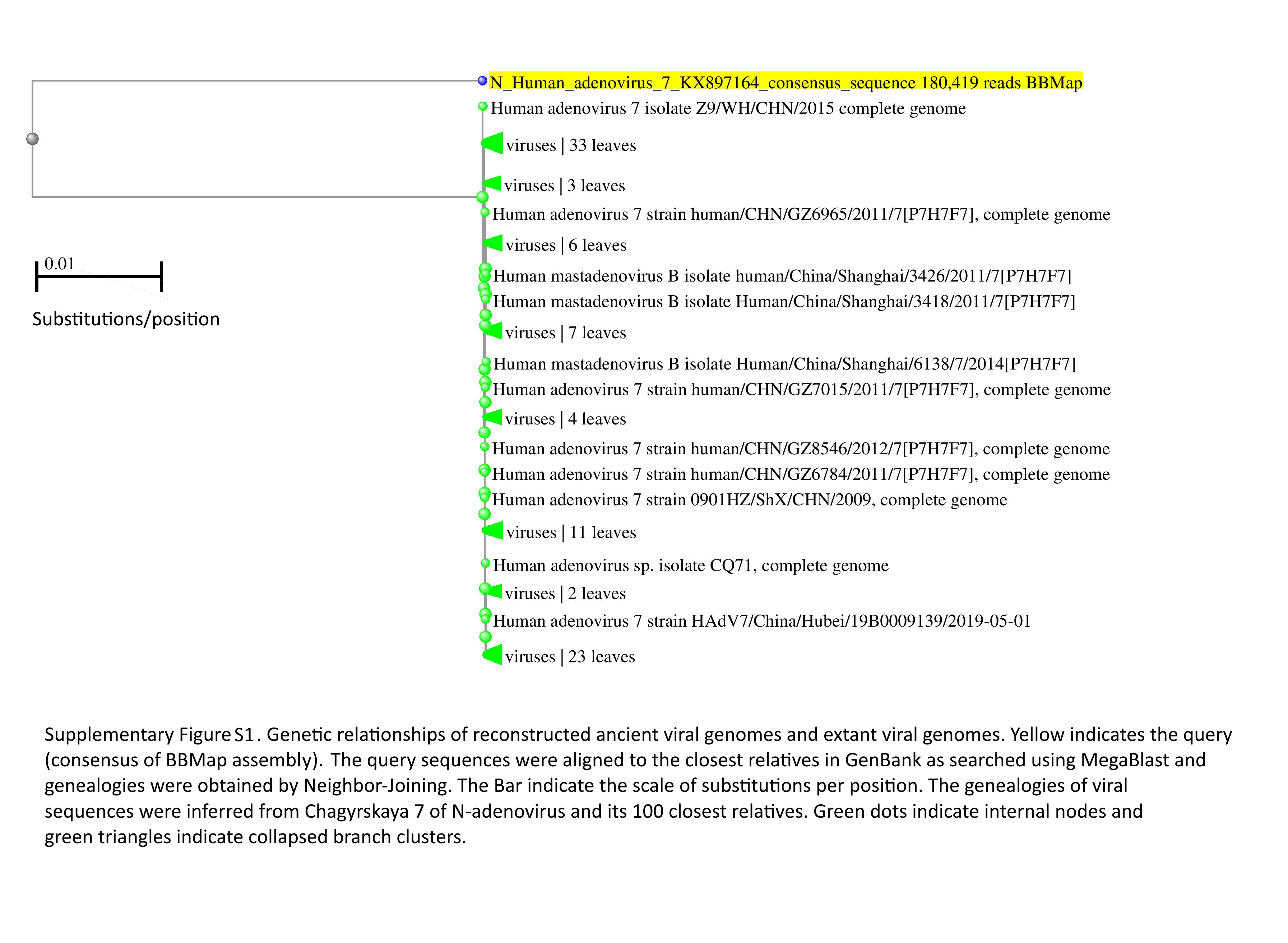

Supplement: Supplementary file 1 [file viruses-16-00856-s001.zip › Supplementary Figure S1 Adenovirus NJ-MegaBlast.png]

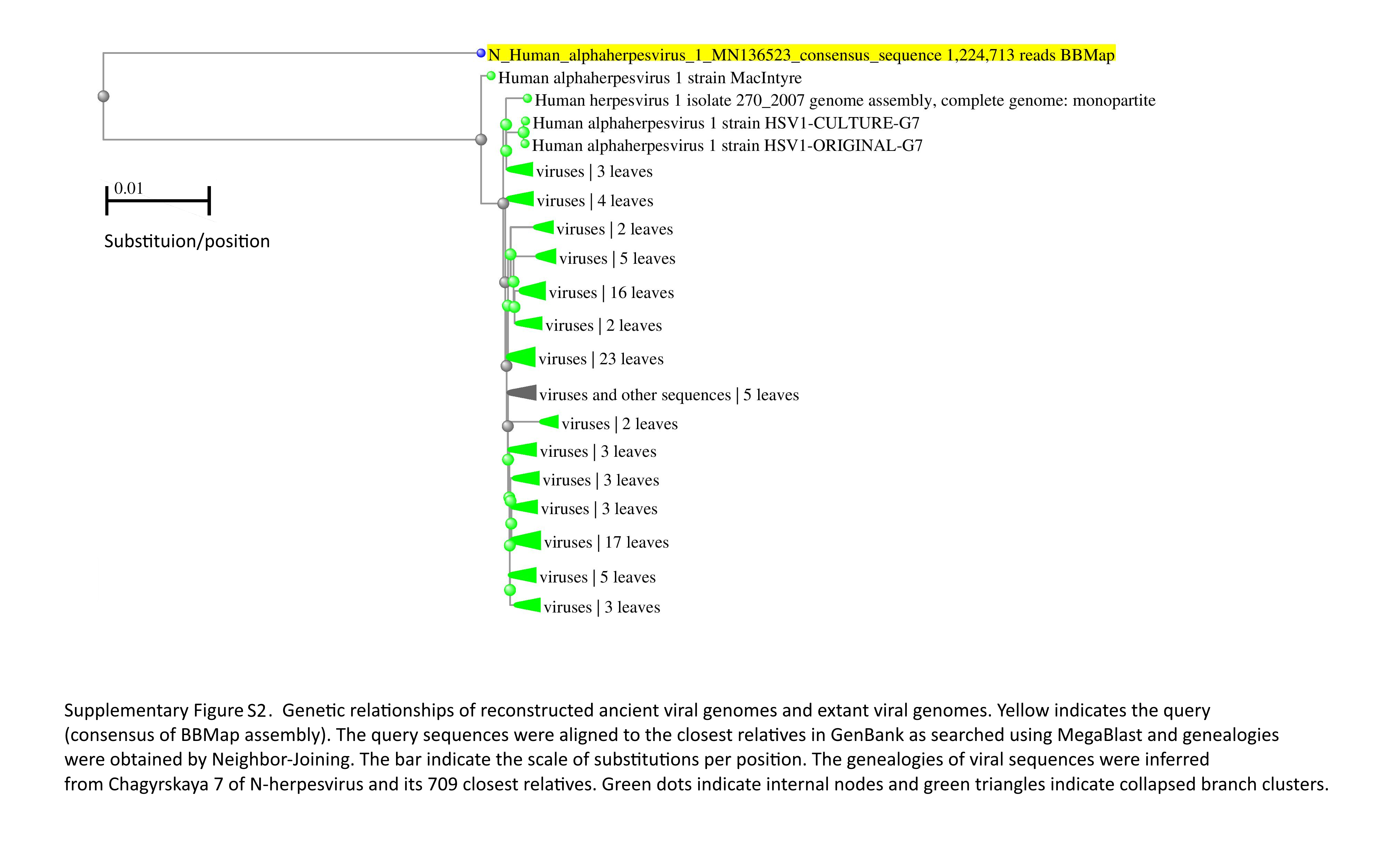

Supplement: Supplementary file 1 [file viruses-16-00856-s001.zip › Supplementary Figure S2 Herpesvirus NJ-MegaBlast.png]

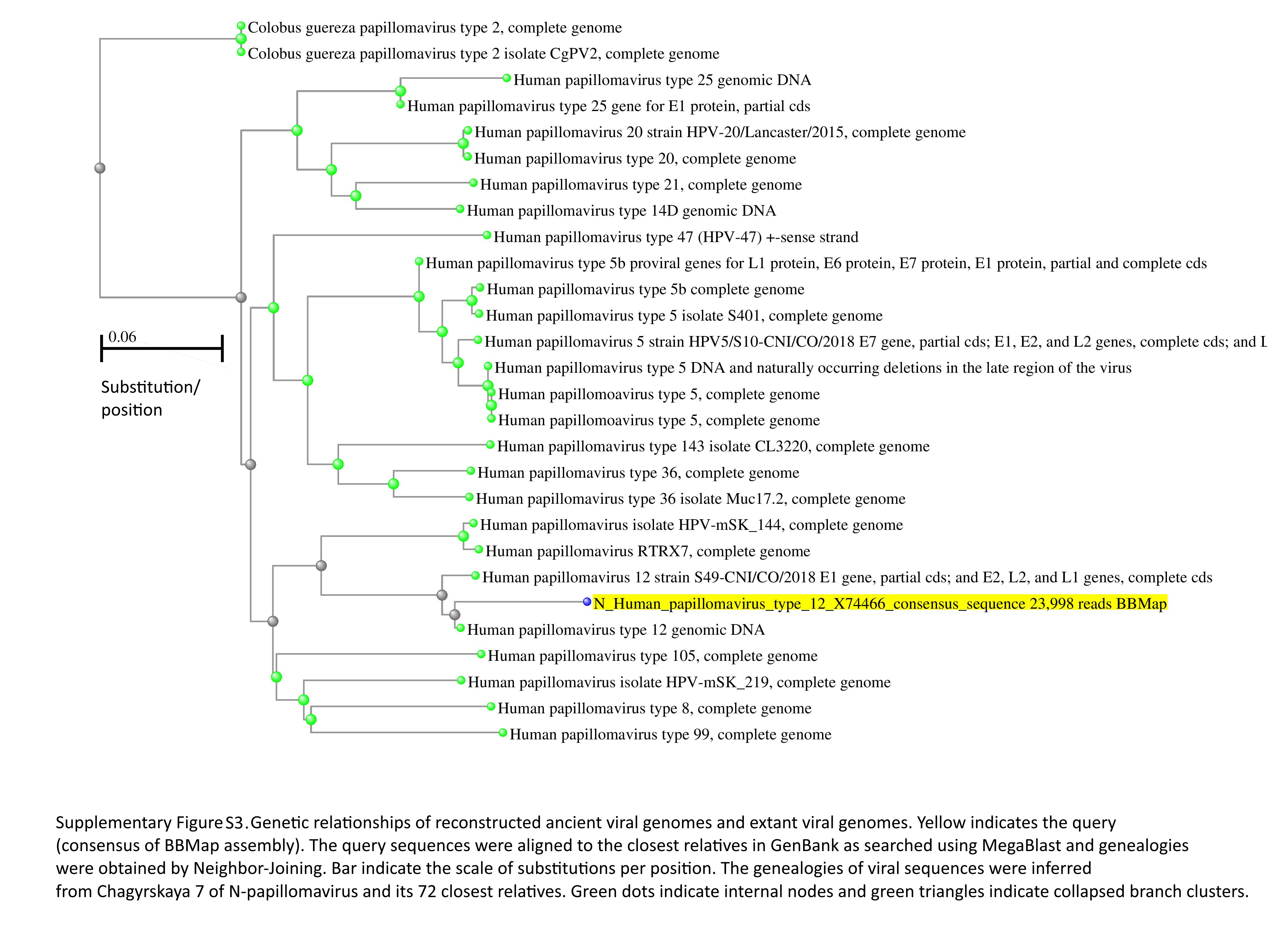

Supplement: Supplementary file 1 [file viruses-16-00856-s001.zip › Supplementary Figure S3 Papillomavirus NJ-MegaBlast.png]

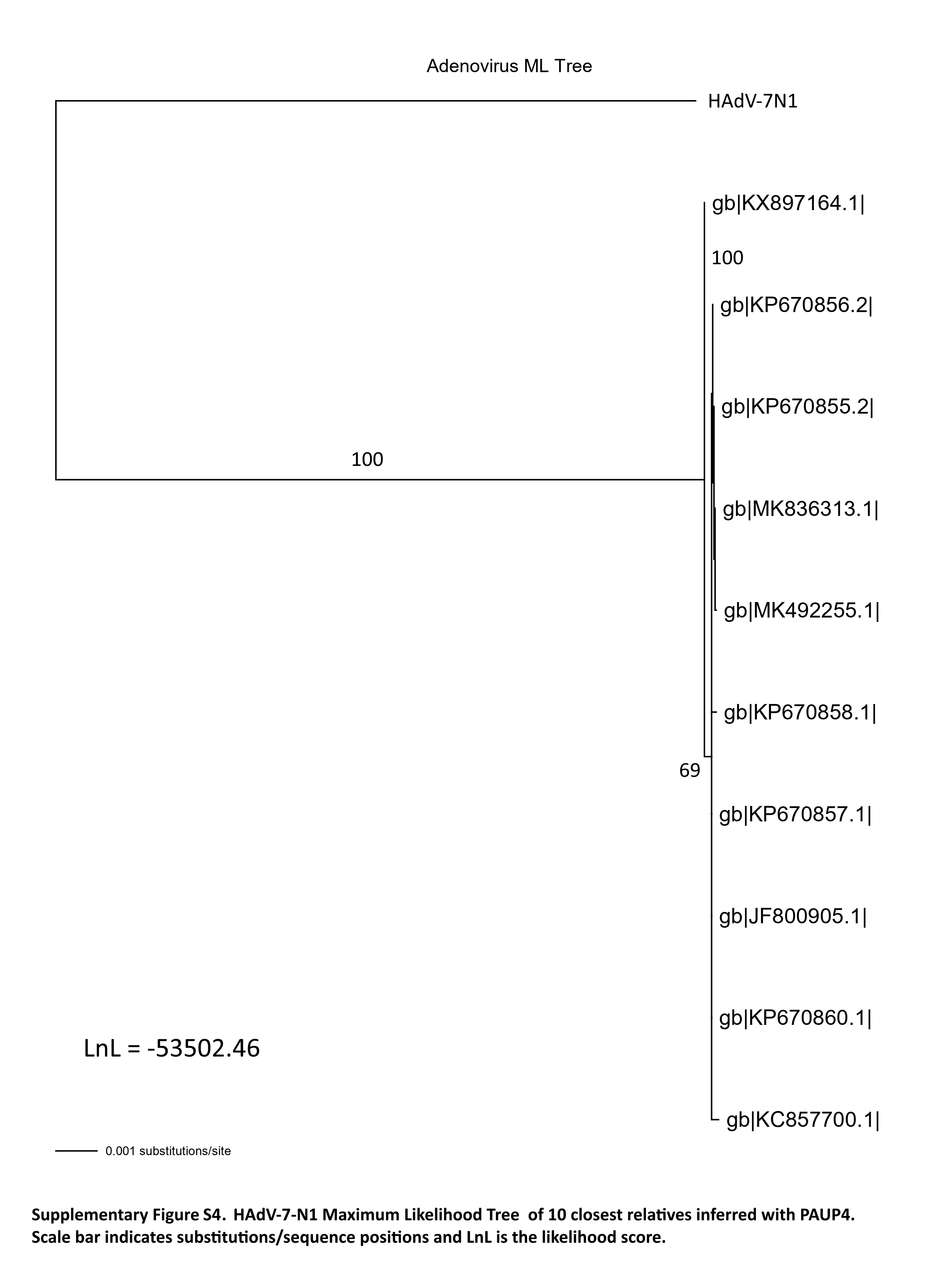

Supplement: Supplementary file 1 [file viruses-16-00856-s001.zip › Supplementary Figure S4 Adenovirus ML Tree.jpg]

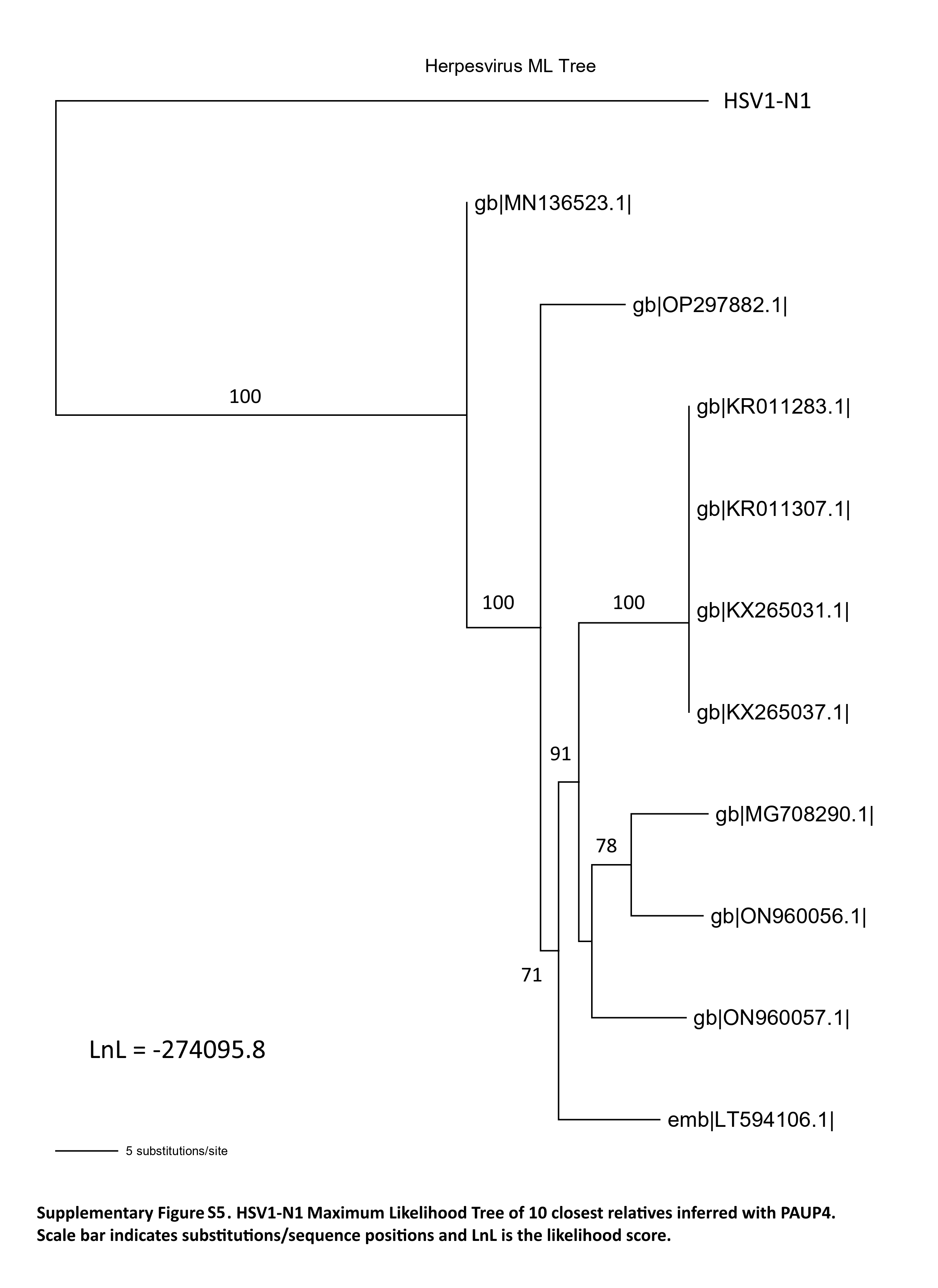

Supplement: Supplementary file 1 [file viruses-16-00856-s001.zip › Supplementary Figure S5 Herpesvirus ML Tree.jpg]

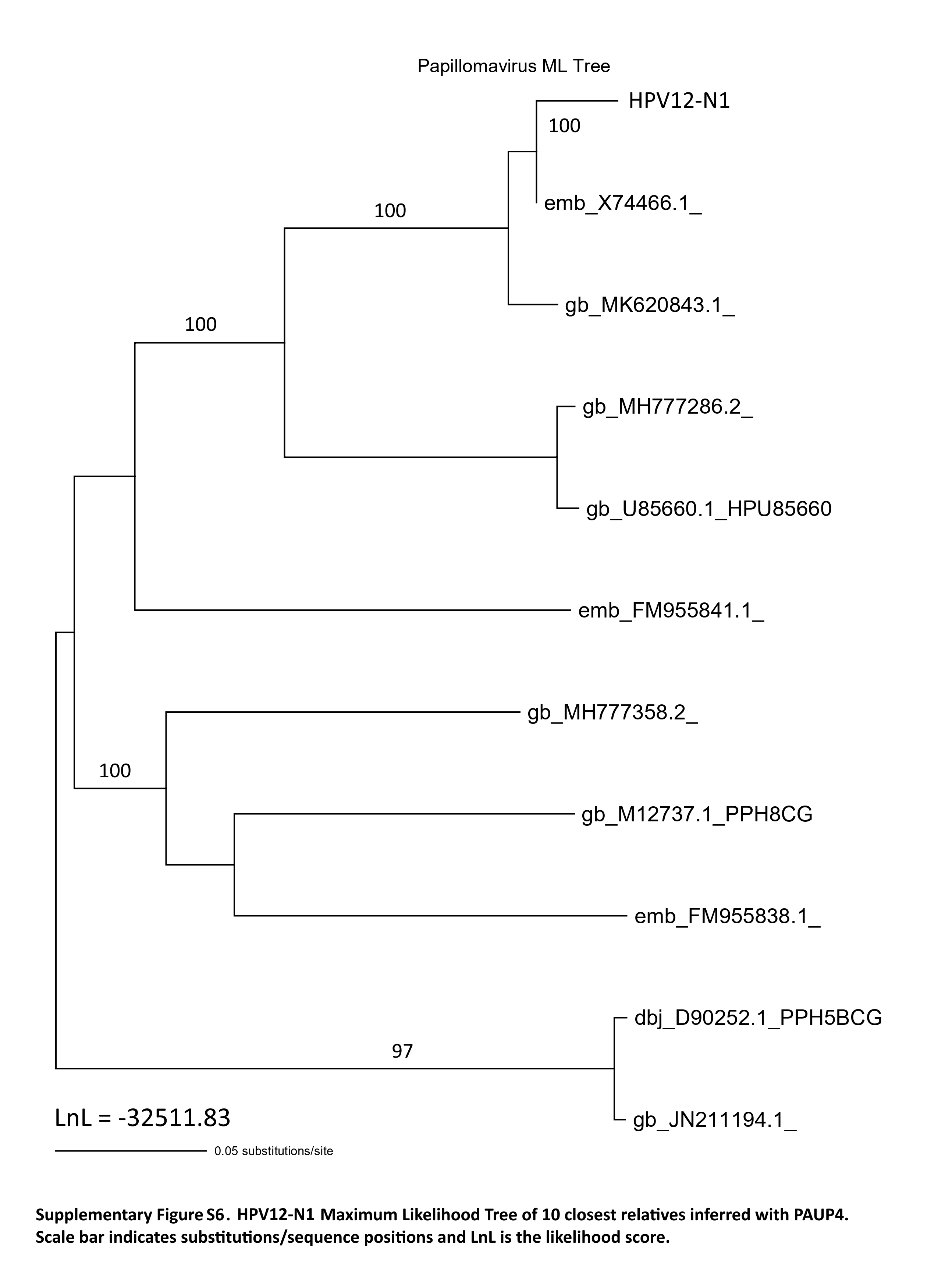

Supplement: Supplementary file 1 [file viruses-16-00856-s001.zip › Supplementary Figure S6 Papillomavirus ML Tree.jpg]
